# Oral Health and the Association with Blood Parameters in Neurogeriatric Inpatients without Relevant Systemic Inflammation: An Observational Study

**DOI:** 10.3390/geriatrics9030055

**Published:** 2024-04-23

**Authors:** Alicia Maria Blasi, Sonja Henny Maria Derman, Asha Kunnel, Pantea Pape, Gabriele Röhrig, Anna Greta Barbe

**Affiliations:** 1 Faculty of Medicine and University Hospital Cologne, Polyclinic for Operative Dentistry and Periodontology, University of Cologne, D-50923 Cologne, Germany; alicia.blasi@uk-koeln.de (A.M.B.); sonja.derman@uk-koeln.de (S.H.M.D.); 2 Clinic for Early Neurological and Interdisciplinary Rehabilitation, St. Marien-Hospital, D-50668 Cologne, Germany; asha.kunnel@cellitinnen.de (A.K.); pantea.pape@cellitinnen.de (P.P.); 3 Department of Health, EUFH-European University of Applied Sciences, D-50996 Cologne, Germany; g.roehrig-herzog@eufh-medica.de

**Keywords:** aged, hospitalization, oral hygiene, periodontitis

## Abstract

As little evidence is available, we report the oral health of neurogeriatric inpatients and the association with hematological parameters representing systemic health. We performed a cross-sectional investigation of 30 patients undergoing neurogeriatric early rehabilitation and excluded systemic inflammation as a trigger for oral infection (C-reactive protein >5 mg/dL). Outcomes included oral health and hygiene status and routine laboratory parameters. Patients (mean age 79 ± 6 years, mean comorbidities 7 ± 3, and mean Barthel Index at hospital admission 31 ± 18) had impaired oral health (mean 18 ± 7 of their own teeth, elevated plaque indices (2.5 ± 0.4), and bleeding on probing (26 ± 17)), representing short- and long-term reduced oral hygiene. Twenty-four (80%) patients had periodontitis. Laboratory parameters for inflammation, nutrition, and anemia did not correlate with oral health parameters (*p* > 0.05). The number of teeth correlated moderately with total protein (Spearman’s rank correlation coefficient (*r_s_*) = 0.524; *p* = 0.003). Plaque indices correlated weakly with number of teeth (*r_s_* = −0.460; *p* = 0.010) and periodontitis diagnosis (*r_s_* = 0.488; *p* = 0.006). Thus, highly vulnerable neurogeriatric inpatients had reduced oral health and hygiene independent of laboratory parameters, representing a high-risk population for oral health problems even without clinically proven systemic infection. This should be considered in future interprofessional therapy planning.

## 1. Introduction

Neurogeriatric patients are geriatric patients with a leading neurological diagnosis [1]. Due to the complexity of neurological dysfunction and the simultaneous presence of multimorbidity, neurogeriatric patients in Germany are treated as inpatients in a hospital setting by a multiprofessional team, in the context of neurogeriatric early rehabilitation. The routine implementation of a multidimensional neurogeriatric assessment allows for the development of an individually adapted treatment plan for every patient [2,3]. However, oral health is not yet an integral component of the multidimensional assessment or routine treatment planning, and it is thus not routinely assessed in hospitalized neurogeriatric inpatients. Consequently, such patients represent a population where little evidence is available regarding their oral health and the association with systemic disease. Recent research supports a link between periodontitis and Alzheimer’s disease, a neurodegenerative disorder that affects millions of people worldwide [4]. It is evident that these diseases share the same or similar modifiable risk factors, but the direction of the link is still under investigation. Some studies indicate that people with periodontitis have a higher risk of developing Alzheimer’s disease [5]. Others have suggested that neurodegenerative diseases may affect a person’s ability to maintain adequate daily oral hygiene, making them more susceptible to developing periodontitis [6]. Yet oral diseases such as oral hygiene deficits, oral inflammation, periodontitis, xerostomia and hyposalivation, carious lesions, and prosthetic rehabilitation needs are highly prevalent in older people in general [7,8]. In addition, studies have revealed poor oral health among patients in acute care hospitals and particularly in rehabilitation medicine [9,10]. A multicenter observational study in elderly rehabilitation patients revealed that more than 70% of participants had oral problems with their teeth, dentures, gums, oral mucosa, tongue, lips, saliva, and swallowing; 30% suffered from 5–7 problems at the same time [10]. This may be due to their acute illnesses but also to pre-existing medical problems that preclude patients from independently maintaining their own oral hygiene. Furthermore, the diagnosis and treatment of acute general diseases, especially in the context of early rehabilitation, is time-consuming for the patient, caregivers, and support staff, which often leads to a rapid breakdown in oral hygiene. However, the gradual deterioration of oral hygiene at home over a longer period of time before hospitalization is an important risk factor for periodontitis [11] and may, in addition to medical consequences such as aspiration pneumonia, lead to a consequent reduction in oral health and oral health-related quality of life [12,13]. All of these issues represent oral frailty, a relatively new construct that has been proposed as a concept for the age-related gradual loss of oral function caused by a range of impairments that worsen daily oral functions, e.g., tooth loss, poor oral hygiene, inadequate dentures, or chewing difficulties associated with age-related changes in swallowing [14]. Oral frailty has been defined as a decline in oral function along with a decline in cognitive and physical functions, such as the oral microbiota and neurodegeneration of Alzheimer’s disease [15].

Multimorbidity is highly prevalent in older people, and symptoms of certain acute or chronic inflammatory diseases may hide or alter other diseases [16], such as oral inflammation. This complicates the quantification of the impact of oral inflammation on their general health. It is also extremely difficult to account for the various heterogeneous influencing factors of oral diseases (especially systemic acute or chronic inflammation) in their analysis, particularly in patient populations with multimorbidity.

Therefore, our observational study aimed to describe the oral health and hygiene of a neurogeriatric inpatient population and to investigate the association with systemic health, as assessed by hematological parameters representing inflammatory status, anemia (e.g., hemoglobin), and nutritional status (e.g., total protein). We included a standardized study population regarding the influencing factor of systemic inflammation by excluding patients with clinical or laboratory-defined acute or chronic inflammation at the time of study inclusion.

## 2. Materials and Methods

### 2.1. Ethics

The study was approved by the local ethics review board of the Medical Faculty of the University of Cologne (no. 20-1721) and was prospectively registered in the German Clinical Trials Register (https://drks.de, accessed on 22 April 2024; DRKS00024446; date of registration 11 June 2021). All methods were performed in accordance with the ethical principles for medical research defined in the Declaration of Helsinki. Written informed consent was provided by every patient before study inclusion and evaluation of recorded medical data. The datasets used and/or analyzed during the current study are available from the corresponding author upon reasonable request.

### 2.2. Study Population

This was a prospective, observational study of patients aged >70 years undergoing early neurological rehabilitation as part of their inpatient hospitalization at the St. Marien-Hospital, Cologne, Germany, between September 2021 and October 2022. Patients without acute or chronic inflammation (hematological C-reactive protein (CRP) ≤ 5 mg/dL) were included. An interdisciplinary board (comprising dentists, a neurologist, and a geriatrician) was established to ensure that only patients with no explanation for low-grade elevated CRP other than oral inflammation were included. Exclusion criteria were as follows: active tumor disease requiring treatment; infection requiring treatment; actual antibiotic treatment; chronic inflammatory disease such as rheumatoid arthritis; chronic inflammatory bowel disease; osteomyelitis; previous (within 2 weeks) apoplectic insult with lysis treatment; glomerular filtration rate < 30 mL/min; dialysis requirement; and coronary heart disease requiring intervention. The first author (A.M.B.) conducted the dental examination at the patient’s bedside during the inpatient stay, using magnifying glasses with light. As no assistant was allowed in the patient room during the COVID-19 pandemic due to visiting regulations, findings were dictated and subsequently documented in writing.

### 2.3. Sample Size Calculation

The case number estimation was designed to detect at least large effects (beta or *r* ≥ 0.5). To achieve a power of 80% at a significance level of at least 5%, a minimum of 26 patients was required. Thus, 30 patients in total were included.

### 2.4. Patient Parameters

Age, gender, general illness, and active pharmaceutical ingredients (APIs) at admission were taken from the patient file. In addition, the Barthel Index (BI) was used to assess performance in activities of daily living at the time of admission. The subdomain ‘grooming’ of the BI refers to personal hygiene and includes brushing teeth and fitting dentures.

### 2.5. Laboratory Parameters

Hematologic parameters were routinely assessed during the inpatient stay. The white blood cell count (WBC) and CRP determined inflammatory status; anemia was evaluated by the red blood cell count, hemoglobin, hematocrit, mean corpuscular volume, mean corpuscular hemoglobin, mean corpuscular hemoglobin concentration, red cell distribution width, ferritin, and transferrin saturation; while the nutritional status was determined by the total protein, albumin, vitamin B12, and folic acid. Anemia was classified according to the World Health Organization definition of anemia [17], with reference hemoglobin values of <12 g/dL in adult females and <13 g/dL in adult males.

### 2.6. Oral Health Parameters

The study design considered parameters that could be assessed at the patient’s bedside without additional diagnostic equipment. For the oral assessment, the total number of teeth, prosthetic situation, and plaque index according to Silness and Loe [18] were documented. A thorough diagnosis of caries may not be possible without the ability to remove biofilm at the patient’s bedside. Therefore, the Decayed, Missing, and Filled Teeth value was not included. To measure subjective dry mouth, patients were asked a yes/no question: ‘Do you feel that your mouth is dry?’. In addition, patients completed a 100 mm visual analog scale (VAS) from 0 mm = ‘not dry at all’ to 100 mm = ‘no saliva at all’. Periodontal status was determined at the oral mesiobuccal and distobuccal sites by 2-point measurement of probing pocket depths (mm), recessions (mm), clinical attachment loss (mm), and bleeding on probing (BOP) as a measure of active inflammation. The maximum degree of furcation involvement and tooth mobility according to the technique recommended by Nyman et al. [19] were documented for each tooth. Based on clinical findings, a periodontal diagnosis was made according to the AAP/EFP (European Federation of Periodontology) new classification for periodontal diseases [20] and according to the previous CDC/AAP classification (Centers for Disease Control and Prevention und American Academy of Periodontology) [21].

### 2.7. Statistical Analysis

Qualitative variables are described by count and percentage. Mean ± standard deviation values were obtained for quantitative variables. Distributions of continuous variables were checked for normality by visual inspection and the Shapiro–Wilk test. Differences between male and female patients for patient and clinical characteristics were tested using Student’s *t*-test. The correlation between periodontal characteristics and laboratory findings was determined using the Spearman rank correlation coefficient *r_s_*, with the significance level set at *p* < 0.05. The correlation coefficients *r_s_* were rated as weak (0.1–0.5), moderate (>0.5–0.8), and strong (>0.8). Linear regression analysis was used to assess possible clinically relevant associations between oral parameters and inflammatory blood values. Therefore, the dependent variables ‘CRP’ and ‘WBC’ were established. Logistic regression analysis was used to assess possible relevant associations between medication intake and subjectively reported dry mouth. Therefore, the dependent variable ‘APIs’ was established. All calculations were performed using IBM SPSS Statistics software, V29 (https://www.ibm.com/products/spss-statistics, accessed on 22 April 2024, RRID:SCR_016479).

## 3. Results

### 3.1. Patient Characteristics

A total of 30 patients (15 male, 15 female; aged 79 ± 6 years) with 7 ± 3 comorbidities and polypharmacy were admitted to the neurogeriatric clinic (Table 1). The most frequent neurological diagnoses were Parkinson’s disease (n = 14, 47%) and polyneuropathy (n = 11, 37%) (Table 2). The mean BI at the time of admission was 31 ± 18; according to the BI subdomain ‘grooming’, 29 (97%) patients were dependent on help with personal care (including brushing teeth). The two-sided *t*-test showed that the only variance between male and female patients was in the BI (*p* = 0.010) (Table 1).

### 3.2. Laboratory Characteristics

The mean red blood cell count (3.86 ± 0.65), hemoglobin (11.70 ± 2.01), hematocrit (35.77 ± 5.43), and total protein (6.08 ± 0.67) were all lower than the reference values, while the mean CRP (1.57 ± 1.08) and ferritin (261.83 ± 212.54) were higher. Other documented blood values were within reference values (Table 3). Anemia was present in 22 (73%) patients, with equal distribution among males and females (Table 4). Due to the heterogeneity of the underlying comorbidities (Table 2) and the resulting variation in active pharmaceutical ingredients among the patients studied, no further investigation of the impact on blood levels in this population was performed.

### 3.3. Oral Health Characteristics

Patients had 18 ± 7 of their own teeth and elevated plaque levels (Plaque Index 2.5 ± 0.4). Fifteen (50%) patients had removable prostheses; of these, three patients did not have their prostheses with them during their inpatient stay. The mean time since the last dental visit was 22 ± 37 (range 1–180) months. Subjective dry mouth was reported by 21 (70%) patients (Table 5). Periodontitis, based on clinical findings according to the AAP/EFP classification, was detected in 24 (80%) patients, of whom seven were diagnosed with severe (stage III) and five with advanced (stage IV) periodontitis (Table 6). No significant differences (all *p* > 0.05) could be found between patients with and without periodontitis in the clinical characteristics examined. Only in the descriptive analysis was the number of comorbidities lower in patients without periodontitis (5.17 ± 3.0) than in patients with periodontitis (7.7 ± 3.4).

### 3.4. Association between Oral Health and Laboratory Parameters

The Plaque Index correlated weakly with transferrin saturation (*r_s_* = 0.383; *p* = 0.037). The number of teeth correlated moderately with total protein (*r_s_* = 0.524; *p* = 0.003). None of the other investigated oral health parameters correlated moderately or strongly with any other investigated laboratory parameter. Linear regression analysis indicated that BOP representing gingival inflammation influenced the WBC (*p* = 0.001). The observed WBC was within the reference range, indicating little or no clinical significance in this population. None of the investigated oral health parameters influenced the laboratory parameter CRP in linear regression analysis.

### 3.5. Association between Oral Health and Patient Parameters

Logistic regression analysis determined that the number of medications did not influence subjective reported dry mouth (*p* = 0.725). Subjective dry mouth VAS values did not correlate with the number of medications.

## 4. Discussion

To our knowledge, this is the first observational study to investigate oral health and hygiene and their association with hematological parameters representing systemic health in a population of neurogeriatric inpatients undergoing early rehabilitation, who had many pre-existing conditions but no relevant acute or chronic inflammation excluded by low levels of C-reactive protein.

The prevalence of anemia in our population was high, as has previously been observed in inpatients [22]. However, the multimorbidity in our patients and the relatively small number of cases did not allow for the clear assignment of the forms of anemia. It was also evident that our patients had reduced oral health and hygiene. This finding is consistent with previous studies which have found that poor oral health, dysphagia, and undernutrition are significantly correlated in geriatric populations [23,24]. There is a high periodontal disease burden in these patients, despite the exclusion of a possible influence of acute or chronic inflammation reflected by hematological C-reactive protein ≤ 5 mg/dL. It was not possible to draw conclusions about potential systemically preventive parameters in this population due to the varying group sizes, with few patients without diagnosed periodontitis according to the AAP/EFP classification and many patients with diagnosed periodontitis. In our population, there was a small group of patients without periodontitis. Although no meaningful comparison analysis between the groups was possible due to the small number, no relevant differences could be found between the groups with regard to the systemic conditions (Barthel Index) and laboratory parameters when the data were viewed descriptively. The only difference was the number of comorbidities, which was smaller in the group without periodontitis. This suggests that healthier patients also had less periodontitis. However, due to the small number of cases, this should be seen as more of an indication. As expected in a population in which relevant systemic inflammation has been ruled out, there was no association between periodontal status (as a marker for oral inflammation) and the laboratory blood parameters used to reflect inflammation. The mouth has been long recognized as a source of systemic infection [25]. Epidemiological evidence is mounting for a bidirectional link (as appears to be true for most diseases involving systemic inflammation) between periodontal disease and various systemic, mainly inflammatory diseases [26]. From a periodontological perspective, our population was in accordance with those in large-scale empirical studies such as the DMS V in Germany [27]. Patients retain many of their own teeth until old age and clinical periodontitis markers are pathologically elevated. In our carefully selected population, CRP was slightly higher than reference values but periodontitis in the clinical setting had no influence on inflammatory blood values. Major systemic inflammation was one of our exclusion criteria; thus, it might be expected that CRP levels would be slightly elevated due to oral inflammation in our population. However, the linear regression analysis did not show any significant influence of the oral health parameters on CRP values. Additionally, the WBC values were within the reference range, despite the significant influence of the oral health parameter BOP in the linear regression. It is possible that immunosenescence and inflammaging may be underlying reasons for the high periodontal disease burden and slightly elevated CRP values observed in our patients, in contrast to those described in younger populations [28,29]. Even in healthy older adults, increased levels of circulating proinflammatory factors such as CRP are detectable compared to young adults [30]. However, this age-related increase is less than 0.5 mg/dL in healthy elderly patients [31]. It is important to note that the total amount of proinflammatory mediators within the body may not reflect the pathological effects of aging [32]. High levels of inflammatory markers have been recorded in centenarians who were otherwise free of major inflammatory diseases. It may be that inflammaging (the chronically increased and dysregulated inflammatory response that is observed with increasing age) is only detrimental if the increase in proinflammatory molecules occurs in anatomical locations where they are able to impair cell and tissue functions [32]. Age-related changes in neutrophils, macrophages, and T cells, for example, result in inflammaging and impaired immune function (immunosenescence) and an inadequate immune response to oral invading pathogens, leading to their chronic persistence within the body [33]. Strict and adequate regulation of the host inflammatory response to oral microbes is required to maintain periodontal health [34]. The summation of these mechanisms and the results in our carefully selected participant group are in accordance the prognosis of shifting the periodontal treatment burden to the later decades of life [35].

In a vulnerable patient population such as ours, the risk of chronic disease goes beyond periodontitis. We found a significant correlation between total protein and number of teeth, which suggests a relationship between protein intake, oral health, and malnutrition in older people, as previously described [36]. The number of own teeth remaining and the periodontal disease load have an influence on chewing function. Structural changes due to periodontitis, such as clinical attachment loss and tooth mobility, reduce chewing efficiency [37]. Nutrition and oral health are inextricably linked. With severely reduced dentition and non-replaced missing teeth, the ability to comminute food decreases [38]. This creates difficulties, especially when ingesting chewing-intensive, protein-rich foods such as meat, and these foods are no longer consumed. Furthermore, particularly in this multimorbid population with high medication intake and other geriatric health issues such as dehydration, oral dryness may be another factor favoring dysphagia and limited chewing function [39,40]. Oral hypofunction and suboptimal nutrition are potential risk factors for physical frailty [41], so it is vital to maintain healthy and functional dentition into old age.

In our patients, the last visit to the dentist was almost 2 years ago and not within the recommended 3–12-month recall interval for routine dental examinations [42]. It is known that with age, control-oriented consultation with the dentist decreases and shifts to the problem-oriented use of dental services. The utilization of dental services is likely to decrease even further in line with increasing frailty and care needs [27]. In our population, studied during September 2021 and October 2022, restrictions due to the COVID-19 pandemic may also have had an impact on attending a control-oriented dental visit [43]. Inadequate daily oral hygiene and the reduction in utilization of dental services are both risk factors for severe periodontitis, increasing its risk by 14.0% [27]. The need for prevention and treatment of the high periodontal disease burden should not be diminished. In vulnerable populations with many pre-existing conditions who now require inpatient care—and who have maintained their own teeth into old age but may not have been under dental care for some time—the hospital can function as an opportunity to identify at-risk patients and enable them to regain access to primary care dental services. Such actions may serve to reduce the disparities in access to dental care observed in this vulnerable patient population. Dentists should therefore be an integral part of interprofessional geriatric care concepts. It is also well known that older people are staying within their own home for longer, especially in Western countries, and the use of dental care is also decreasing [44]. Here too, a hospital stay could be a suitable interface to re-initiate dental care. The development of future concepts is certainly necessary to define responsibility but also to scientifically examine suitable validated and standardized assessment instruments.

Our study evaluated a patient population that is rarely accessed in general dental practice. As in every observational study, it has some limitations. Only the influence of oral health parameters on routine laboratory blood values was considered. It was not possible to determine the influence on other laboratory parameters. We were unable to determine a link between periodontitis and clinical laboratory parameters, but this does not mean that associations do not exist. Due to the lack of a control group and lack of an analysis to rule out confounding variables due to data heterogeneity, the data should be interpreted with caution. In future studies, additional endpoints and the comparison to healthy subjects would certainly be necessary to better understand the complex overall influences of multimorbid patients.

## 5. Conclusions

This study indicates that neurogeriatric inpatients, as a heterogeneous group, experience prolonged periods without dental care. This puts them at high risk of developing oral health and hygiene issues such as dental plaque, periodontitis, and active inflammation. In this rarely described population with minimal systemic inflammatory influences, we were unable to detect the expected negative impact of their oral health and hygiene problems (such as periodontitis) on hematological parameters representing inflammatory status (CRP and WBC), anemia (red blood cell count, hemoglobin, hematocrit, mean corpuscular volume, mean corpuscular hemoglobin, mean corpuscular hemoglobin concentration, red cell distribution width, ferritin, and transferrin saturation), and nutritional status (total protein, albumin, vitamin B12, and folic acid). This should be interpretated with caution due to the cross-sectional analysis and the high variability of data, especially with regard to clinical characteristics. Since the data show that dental care for this population is not meeting their documented needs, the availability of dental expertise may provide a solution. The integration of dentists into the multiprofessional care of neurogeriatric inpatients would be a sensible approach.

## Figures and Tables

**Table 1:** Patient and clinical characteristics.

Characteristic	Total (N = 30)
Age (years)	79 ± 6 (70–90)
Comorbidities (n)	7 ± 3 (1–18)
APIs (n)	8 ± 4 (2–16)
BI (at admission)	31 ± 18 (10–75)
BI subdomain grooming needs help with personal care, n (%)	29 (97)

Results are mean ± standard deviation (range), unless otherwise shown. Abbreviations: APIs, active pharmaceutical ingredients; BI, Barthel Index.

**Table 2:** Distribution of comorbidities detected in two or more patients.

Comorbidity	n (%)
Hypertension	22 (73)
Parkinson’s disease	14 (47)
Polyneuropathy	11 (37)
Vitamin B12 deficiency	9 (30)
Hypothyroidism	7 (23)
Sarcopenia	7 (23)
Atrial fibrillation	6 (20)
Coronary heart disease	6 (20)
Folate deficiency	5 (17)
Dementia	5 (17)
Type 2 diabetes mellitus	5 (17)
Depression	5 (17)
Osteoporosis	4 (13)
Iron deficiency anemia	4 (13)
Hyponatremia	3 (10)
Obesity	3 (10)
Chronic obstructive pulmonary disease	2 (7)
Stasis dermatitis	2 (7)
Hyperuricemia	2 (7)
Hypercholesterolemia	2 (7)
Heart failure	2 (7)
Bronchial asthma	2 (7)

**Table 3:** Laboratory chemical blood analysis.

Laboratory Parameter	Reference Values	Mean ± SD ^1^
WBC count (n/nL)	4.0–10.0	6.64 ± 1.72
CRP (mg/dL)	<0.4	**1.57 ± 1.08**
RBC count(n/pL)	4.4–5.9	**3.86 ± 0.65**
Hemoglobin (g/dL)	13.5–17.5	**11.70 ± 2.01**
Hematocrit (%)	40-53	**35.77 ±5.43**
MCV (fL)	82–98	92.93 ± 7.07
MCH (pg)	27–33	30.40 ± 2.85
MCHC (g/dL)	31–36	32.63 ± 1.54
RDW (%)	11.6–16.0	14.30 ± 1.62
Ferritin (ng/mL)	20–250	**261.83 ± 212.54**
Transferrin (mg/dL)	200–360	202.47 ± 46.80
Transferrin saturation (%)	16–45	23.73 ± 15.79
Total protein (g/dL)	6.4–8.3	**6.08 ± 0.67**
Albumin (mg/dL)	3500–5200	3641 ± 570
Vitamin B12 (pg/L)	197–771	641.77 ± 325.29
Folic acid (pg/mL)	>3.9	8.64 ± 6.25

^1^ Bold: not within the reference values. Abbreviations: CRP, C-reactive protein; MCH, mean corpuscular hemoglobin; MCHC, mean corpuscular hemoglobin concentration; MCV, mean corpuscular volume; RBC, red blood cell; RDW, red cell distribution width; SD, standard deviation; WBC, white blood cell.

**Table 4:** Anemia among inpatients according to World Health Organization (WHO) criteria.

WHO Criteria for Anemia	Neurogeriatric Inpatients, n (%)		
	Total	Males	Females
Hemoglobin <13 g/dL in males, <12 g/dL in females	22 (73)	11 (73)	11 (73)

**Table 5:** Oral health characteristics.

Characteristics	Neurogeriatric Inpatients (N = 30)
Teeth (n)	18 ± 7 (5–28)
Plaque Index	2.5 ± 0.4 (1.6–3.0)
Patients with removable prosthesis, n (%)	15 (50)
Last dental visit (months)	22 ± 37 (1–180)
Subjective reported dry mouth, n (%)	
No	9 (30)
Yes	21 (70)
Xerostomia VAS (mm)	51.4 ± 22.6 (0–100)

Results are mean ± standard deviation (range), unless otherwise shown. Abbreviation: VAS, visual analog scale.

**Table 6:** Periodontal clinical characteristics.

Characteristics	Neurogeriatric Inpatients (N = 30)
Mean PPD (mm)	3.1 ± 0.7 (2.2–4.9)
Maximal PPD (mm)	5.6 ± 2.4 (3–14)
Mean CAL (mm)	4.7 ± 1.9 (2.3–8.6)
BOP (%)	26 ± 17 (7–69)
Tooth mobility index	0.8 ± 1.2 (0–3)
Degree of furcation	0.7 ± 1.0 (0–3)
Periodontal Staging (AAP/EFP), n (%)	
Stage I	4 (13)
Stage II	8 (27)
Stage III	7 (23)
Stage IV	5 (17)
No periodontitis	6 (20)
Extent of PA in stage III and IV, n (%)	
Localized	1 (8)
Generalized	11 (92)
Periodontal case definition (CDC/AAP), n (%)	
No periodontitis	5 (17)
Mild periodontitis	10 (33)
Moderate periodontitis	7 (23)
Severe periodontitis	8 (27)

Results are mean ± standard deviation (range), unless otherwise shown. Abbreviations: AAP, American Academy of Periodontology; BOP, bleeding on probing; CAL, clinical attachment loss; CDC, Centers for Disease Control and Prevention; EFP, European Federation of Periodontology; PPD, probing pocket depth; SD, standard deviation.

## Data Availability

The datasets used and/or analyzed during the current study are available from the corresponding author on reasonable request.
